# Multiple Time Intervals of Visual Events Are Represented as Discrete Items in Working Memory

**DOI:** 10.3389/fpsyg.2018.01340

**Published:** 2018-08-02

**Authors:** Zhiwei Fan, Yuko Yotsumoto

**Affiliations:** Department of Life Sciences, The University of Tokyo, Tokyo, Japan

**Keywords:** time intervals, working memory, memory load, serial position, similarity, signal detection theory

## Abstract

Previous studies on time perception and temporal memory have focused primarily on single time intervals; it is still unclear how multiple time intervals are perceived and maintained in working memory. In the present study, using Sternberg’s item recognition task, we compared the working memory of multiple items with different time intervals and visual textures, for sub- and supra-second ranges, and investigated the characteristics of working memory representation in the framework of the signal detection theory. In Experiments 1–3, gratings with different spatial frequencies and time intervals were sequentially presented as study items, followed by another grating as a probe. Participants determined whether the probe matched one of the study gratings, in either the temporal dimension or in the visual dimension. The results exhibited typical working memory characteristics such as the effects of memory load, serial position, and similarity between probe and study gratings, similarly, to the time intervals and visual textures. However, there were some differences between the two conditions. Specifically, the recency effect for time intervals was smaller, or even absent, as compared to that for visual textures. Further, as compared with visual textures, sub-second intervals were more likely to be judged as remembered in working memory. In addition, we found interactions between visual texture memory and time interval memory, and such visual–interval binding differed between sub- and supra-second ranges. Our results indicate that multiple time intervals are stored as discrete items in working memory, similarly, to visual texture memory, but the former might be more susceptible to decay than the latter. The differences in the binding between sub- and supra-second ranges imply that working memory for sub- and supra-second ranges may differ in the relatively higher decision stage.

## Introduction

Time perception is essential for the survival of animals and humans, and that in the scale of hundreds of milliseconds to minutes is crucial for many important conscious behaviors (Buhusi and Meck, 2005. It inherently involves working memory (Gibbon et al., 1984) or shares common mechanisms with it (Gu et al., 2015). As a stimulus persists, the time interval defined by the stimulus needs to be constantly updated in working memory. In some cases, especially for human activities, it may be necessary to remember several time intervals simultaneously in working memory.

However, it is still unclear how time intervals are stored in and retrieved from working memory. Previous studies on time have mainly focused on the perception of single time intervals, and they adopted measures such as verbal estimation, reproduction, production, and comparison (Grondin, 2010; Wearden, 2016). Only a few studies have examined the perception and memory of multiple time intervals. Some recent studies have stressed the important role of working memory in perceptual timing tasks and they have examined working memory for multiple time intervals of auditory events (Teki and Griffiths, 2014, 2016; Manohar and Husain, 2016). However, these studies used the time reproduction task, yielding responses as a continuous variable, that is, reproducing the time interval as a quantity instead of recognizing it as an object or an item. Thus, time was quantitatively measured as a continuous magnitude rather than being qualitatively recognized as discrete items, which weakened the claims of these studies that time intervals can be stored as distinct items in working memory. Furthermore, temporal working memory has not yet been directly compared with working memory for non-temporal modalities. Thus the nature of working memory for time intervals remains unknown.

In the present study, we used Sternberg’s item recognition task (Sternberg, 1966) to examine working memory for time intervals, and compared its characteristics with those for visual objects. Sternberg’s item recognition task is a widely used paradigm for studying working memory for items. In the task, several items (study items) are sequentially presented and remembered, and then they are compared with a final item (probe) after a short delay. The participants are expected to indicate whether the probe is a target (the same as one of the study items) or a lure (not the same as any of the study items). The working memory performance on the recognition task can be explained using the signal detection theory (SDT) (Macmillan and Creelman, 2005). The SDT explains how well the items are perceptually discriminated between targets and lures, measured by sensitivity (d′), and what criterion is used to determine if a particular item is the target or the lure, measured by decision criterion (C). The d′ reflects a relatively earlier (e.g., sensory) stage of processing, and the C reflects a later (decision) stage of processing. Modeling item recognitions using SDT allows independent assessments of the two stages of processing (Rotello, 2017). When the SDT is incorporated into the recognition task, a receiver operating characteristic (ROC) curve can be derived from plotting hit rate (the proportion of correct recognitions of targets) as a function of false-alarm rate (the proportion of recognizing lures as a target). The ROC curve reflects a constant d′ that represents discriminability and a variable C that represents response bias.

One key characteristic variable of working memory in Sternberg’s task is memory load (or set size, list length) (Sternberg, 1966). Specifically, the memory performance worsens with an increase in the memory load, that is, an increase in the number of study items. This memory load effect indicates limited working memory capacity for items. For visual items, there are two competing views on working memory capacity. One perspective proposes that items may be stored in a fixed number of slots with a fixed resolution (Luck and Vogel, 1997; Zhang and Luck, 2008), whereas the other proposes that the working memory resource can be flexibly distributed across all items (Bays and Husain, 2008; Ma et al., 2014). Despite the difference, both views are related to the discrete item-based representations (Xie and Zhang, 2017), that is, both views at least agree that visual objects are represented as discrete items in visual working memory, and that memory load influences the memory performance for visual items. Therefore, if time intervals are represented as items similar to the visual objects, there should also be a memory load effect for the temporal items. To clarify, the term “discrete” used in this study corresponds to the item representation, that is, being represented individually and recognizable, different from what is often used to describe the “slot” property of working memory storage (Luck and Vogel, 1997; Zhang and Luck, 2008).

Another prominent characteristic variable for visual working memory is the serial position of an item on the study list (Oberauer, 2003). The serial position effect refers to the better recall or recognition of the first or last presented items than for the items presented in the middle. This effect contains two types: one is the primacy effect (the performance is better for the first or first few items presented), and the other is the recency effect (the performance is better for the last or last few items presented). Note that the recognition of visual items often yields no primacy effect, but only exhibits a last-item recency effect (e.g., Phillips and Christie, 1977; Avons, 1998).

The similarity between items is also an important variable in visual working memory for multiple items (Jiang et al., 2016). The noisy exemplar model of memory (Kahana and Sekuler, 2002) represents visual items as coordinates with noise in a multi-dimensional feature space. Similarity is defined as the reciprocal of the Euclidian distance between two items in this multi-dimensional space. For a probe that does not match any of the study items, the larger the sum of similarities between the probe and each of the study items, the higher is the possibility that it would wrongly be judged as the target. Whether the probe is judged as the target or the lure is determined by whether the summed similarity crosses a decision criterion (Nosofsky, 1984; McKinley and Nosofsky, 1996; Kahana and Sekuler, 2002). The summed similarity requires the discrete representations of items before being computed (Zhou et al., 2004). If time intervals are represented discretely, the judgment of lure intervals should be subject to the summed similarity as well.

Further, the item representations of visual objects are related to binding (Burwick, 2014). Binding integrates different visual features (e.g., color and shape) into a distinctive object (e.g., a red triangle) so that they can be recognized as a distinctive item. Therefore, if time intervals are represented as items, the temporal features may be subject to binding. A recent study provided evidence suggesting that time intervals can be bound into auditory event representations (Bogon et al., 2017). On the other hand, however, it remains unclear if the recognition of temporal features is also affected by visual features.

In the present study, we hypothesized that, similarly, to visual objects, time intervals can be stored as discrete items in working memory. The presence of the effects of memory load, serial position, and similarity for time intervals would provide support for this hypothesis. The possible binding of temporal and visual features, which may be the underlying mechanism of item representations of time intervals, was also investigated. In addition, previous studies suggested that time intervals are more modality specific for the sub- than for the supra-second range (Rammsayer et al., 2015; Mioni et al., 2016). The processing of these two ranges of time intervals may be influenced by visual features in different ways. Therefore, we also compared working memory for time intervals in sub- and supra-second ranges.

## Experiment 1

In Experiment 1, we measured working memory for visual textures and for intervals in the sub-second range, and compared the visual working memory with temporal working memory. The effects of memory load, serial position, and similarity were examined to evaluate the hypothesis.

### Materials and Methods

#### Participants

Sixteen naïve volunteers from The University of Tokyo (7 females, mean age: 22 years, range: 19–28 years) participated in Experiment 1, after excluding one participant whose average accuracy of recognition did not exceed the chance level.

In all experiments, the participants gave written informed consent to participate in the experiment in accordance with the Declaration of Helsinki. The protocol was approved by the Institutional Review Boards of The University of Tokyo, and all experiments were carried out in accordance with the guidelines set by the Ethics Committee of The University of Tokyo. All participants reported having normal or corrected-to-normal vision.

#### Stimuli

Stimuli for each trial were drawn from a pool of two-dimensional (2D) sinusoidal gratings that are often employed in studying recognition/working memory (e.g., Kahana and Sekuler, 2002; Yotsumoto et al., 2008). Gratings were of 25 different spatial frequencies, derived by combining five vertical and five horizontal frequencies of 1.51, 1.75, 2, 2.25, and 2.49 cycles per degree (CPD), similar to those used by Yotsumoto et al. (2008). The gratings were presented at 10 different time intervals from 0.25 to 0.97 s, with a linear increment of 0.08 s. The numbers of different visual or temporal stimuli rendered the probe prediction impossible. The luminance profile of the gratings was as follows:

$$L(x,y)={L_{avg}}^{*}(1+c^{*}(\cos(2^{*}\pi^{*}f_{x}^{*}x)+\cos\left(2^{*}\pi^{*}f_{y}^{*}y\right))/2),$$

where L*_avg_* is the mean luminance, of 20 cd/m^2^; c is the contrast; *f_x_* is the spatial frequency of the vertical fundamental component, in CPD; and *f_y_* is the spatial frequency of the horizontal fundamental component. The contrast was set at 1, which was well above the detection threshold. The gratings were five degrees wide and they were windowed by a 2D Gaussian function with a space constant of one degree. The stimulus parameters were determined with an intention to equalize task difficulties between the interval session and the visual texture session.

Working memory performance for time intervals and for visual textures were measured in two separate sessions. In the interval session, participants judged the time intervals while ignoring the visual textures, and in the texture session, participants judged the visual textures while ignoring the time intervals. Identical sets of stimuli were used in both sessions. For each trial in each session, the study items were either one, two, or three gratings, yielding three memory loads. The spatial frequencies of the study items were randomly selected from the 25 different spatial frequencies forming different visual textures, and the time intervals of the study items were randomly selected from the 10 different time intervals. Another item was drawn as the probe, with the following constraints: in a target trial, the probe matched one of the study items, and the serial position in each memory load was probed equally frequently; and in a lure trial, the probe did not match any of the study items. The lure probe was also set such that it was not the neighbor of any of the study items in terms of magnitude, for either visual textures or time intervals. This was to control the similarity between the lure probes and the study items, and to make the lure probes distinguishable from the study items. In the interval session where participants judged the time intervals while ignoring the visual textures, the visual textures served as context features. In the texture session where participants judged the textures while ignoring the time intervals, the time intervals served as context features. When the context feature of the probe matched the context feature of the study item, the context was considered to be “repetition,” and when the context feature did not match the study item, the context was considered to be “switch.”

#### Apparatus

Stimuli were generated on a desktop computer (HP Compaq 8200 Elite), using MATLAB 2014a (The MathWorks, Inc., Natick, MA, United States) with Psychophysics Toolbox Version 3 (Brainard, 1997; Pelli, 1997; Kleiner et al., 2007), and they were presented on a 22-inch CRT monitor (Diamondtron M^2^ RDF223H, MITSUBISHI, Tokyo, Japan) with a refresh rate of 85 Hz and a resolution of 1,024 pixels × 768 pixels. The monitor was gamma calibrated by a ColorCAL MKII colorimeter (Cambridge Research Systems Ltd., Rochester, Kent, United Kingdom). The experiment was conducted in a dark room. Participants sat in front of the monitor, at a distance of 57.3 cm, with their heads on a chin rest.

#### Procedure

**FIGURE 1:**
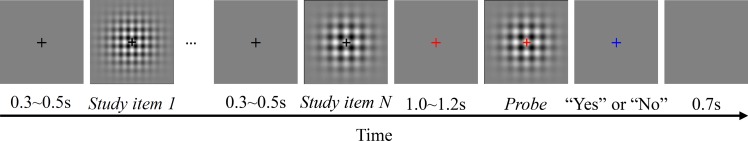
Time course of the recognition/working memory task in Experiments 1 and 3. The number of study items (N) was either one, two, or three in Experiment 1, and was always three in Experiment 3.

All participants completed two sessions. The order of the sessions was counterbalanced across participants. Participants were instructed to refrain from verbally counting (Rattat and Droit-Volet, 2012). **Figure 1** shows the time course of a typical trial in a session. Each trial began with a black fixation cross presented at the center of the screen for 0.3–0.5 s, followed by N (1, 2, or 3) gratings with different visual textures and time intervals, consecutively presented as study items. If *N* > 1, the black fixation cross appeared again for 0.3–0.5 s as an inter-stimulus interval (ISI), followed by another study item. During the presentation of the study items, the fixation cross remained black. The cross then turned red for 1.0–1.2 s, and finally, the probe grating appeared. After the probe was presented, the cross turned blue. Participants were asked to answer whether the probe matched one of the study items by pressing a button assigned to “Yes” or “No.” The right and left arrow keys were used to register the responses. The participants used their right index finger to make the responses. For half of the participants, the right and left arrow keys were assigned to “Yes” and “No,” respectively, while the keys were reversed for the other half of the participants. Additionally, participants were asked to respond as quickly as possible, without sacrificing accuracy. After the response was registered, the trial was ended and it was followed by a new trial after an inter-trial interval (ITI) of 0.7 s.

Each session consisted of 24 practice trials and 360 test trials composed of a combination of factors as memory load (number of study items: 1, 2, or 3), serial position (1 for a memory load of 1; 1 or 2 for a memory load of 2; 1, 2, or 3 for a memory load of 3), context (repetition or switch) and trial type (target trials or lure trials).

### Results

Individual trials associated with response times (RTs) beyond three standard deviations from the mean RT were removed from further analyses. The same exclusion criteria were also employed in Experiments 2 and 3. The average accuracy across all available participants for the time intervals was 65.93 ± 5.70%, for the visual textures was 72.96 ± 6.69%; the group RT across all available participants for the time intervals was 0.86 ± 0.39 s, and for the visual textures was 0.58 ± 0.24 s.

#### Memory Load Effect

Besides the proportion of correct responses, in order to reveal the underlying processes of the memory load effect in the SDT framework, we also calculated the sensitivity (d′) and decision criterion (C). The magnitude of d′ reflects how well participants perceptually discriminated between targets and lures. The sign of the C indicates the bias of participants’ decisions, with a negative sign corresponding to the response bias toward answering “Yes” (targets) and a positive sign corresponding to the response bias toward answering “No” (lures) (Macmillan and Creelman, 2005).

**FIGURE 2:**
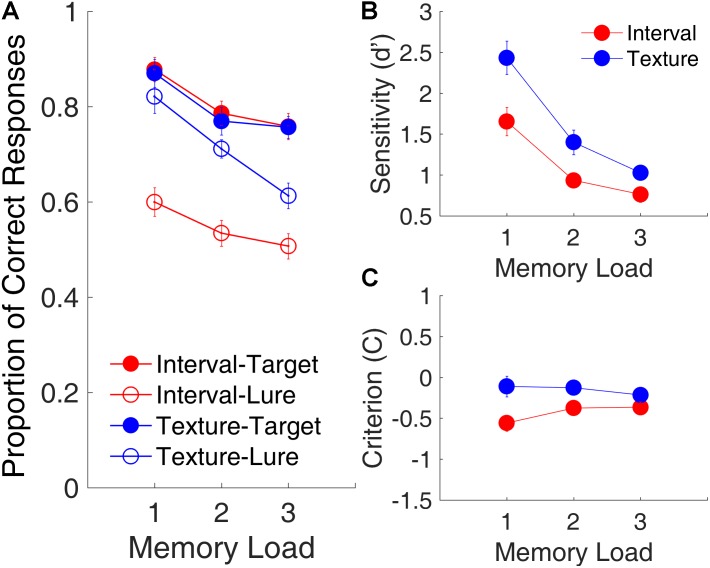
Memory load effects in Experiment 1. **(A)** Proportion of correct responses as a function of memory load. Filled and open circles represent target and lure trials, respectively. **(B)** d′, as a function of memory load. **(C)** C, as a function of memory load. Red and blue represent interval and texture trials, respectively. Error bars represent ± 1 standard error of the mean.

**Figure 2** shows the effects of memory load on the proportion of correct responses, d′, and C plotted across three memory loads and collapsed across serial positions. A repeated-measures ANOVA with three factors [memory load, modality (time intervals or visual textures), and trial type (lure or target)] was conducted on the proportion of correct responses. Results revealed the main effects of memory load [*F*_(2,30)_ = 77.30, *p* < 0.001, $\eta_{p}^{2}$ = 0.838, Greenhouse-Geisser (GG) corrected for non-sphericity], modality [*F*_(1,15)_ = 55.21, *p* < 0.001, $\eta_{p}^{2}$ = 0.723, GG corrected], and trial type [*F*_(1,15)_ = 39.17, *p* < 0.001, $\eta_{p}^{2}$ = 0.786, GG corrected]; and a significant interaction of modality and trial type [*F*_(1,15)_ = 21.72, *p* < 0.001, $\eta_{p}^{2}$ = 0.592, GG corrected]. The main effect of memory load indicated that memory performance declined significantly as memory load increased. The interaction of modality and trial type revealed that the differences between interval memory and texture memory differed between the targets and the lures.

Further, a repeated-measures ANOVA with two factors (memory load and modality) on the proportion of correct responses showed only a main effect of memory load for the target trials [*F*_(2,30)_ = 24.68, *p* < 0.001, $\eta_{p}^{2}$ = 0.622]. For the lure trials, in addition to a main effect of memory load [*F*_(2,30)_ = 17.90, *p* < 0.001, $\eta_{p}^{2}$ = 0.544], there was a main effect of modality [*F*_(1,15)_ = 73.55, *p* < 0.001, $\eta_{p}^{2}$ = 0.831] and a significant interaction between memory load and modality [*F*_(2,30)_ = 5.10, *p* = 0.012, $\eta_{p}^{2}$ = 0.254]. The main effect of modality for lure trials, that is, the proportion of correct responses, was significantly lower for lure intervals than for lure textures, suggesting that participants tended to misjudge the lure intervals as targets more often than they tended to misjudge the lure textures as targets.

A repeated-measures ANOVA with two factors (memory load and modality) on d′ showed main effects of memory load [*F*_(2,30)_ = 89.72, *p* < 0.001, $\eta_{p}^{2}$ = 0.857, GG corrected] and modality [*F*_(1,15)_ = 25.72, *p* < 0.001, $\eta_{p}^{2}$ = 0.632, GG corrected], and a significant interaction of memory load and modality [*F*_(2,30)_ = 4.61, *p* = 0.032, $\eta_{p}^{2}$ = 0.235, GG corrected]. The main effect of modality indicated that, despite our intention to equalize task difficulties between the interval and texture sessions, d′ was smaller for time intervals than for visual textures, indicating that the discriminability between targets and lures was better in the texture task than it was in the interval task. The same ANOVA on C showed a main effect of modality [*F*_(1,15)_ = 20.30, *p* < 0.001, $\eta_{p}^{2}$ = 0.575, GG corrected] and a significant interaction of memory load and modality [*F*_(2,30)_ = 7.91, *p* = 0.005, $\eta_{p}^{2}$ = 0.345, GG corrected]. The main effect of modality indicated that C was more negative for time intervals, suggesting that participants were more biased when they judged time intervals as targets than when they so judged visual textures. This finding was consistent with the lower proportion of correct responses for the lure trials.

#### Serial Position Effect

**FIGURE 3:**
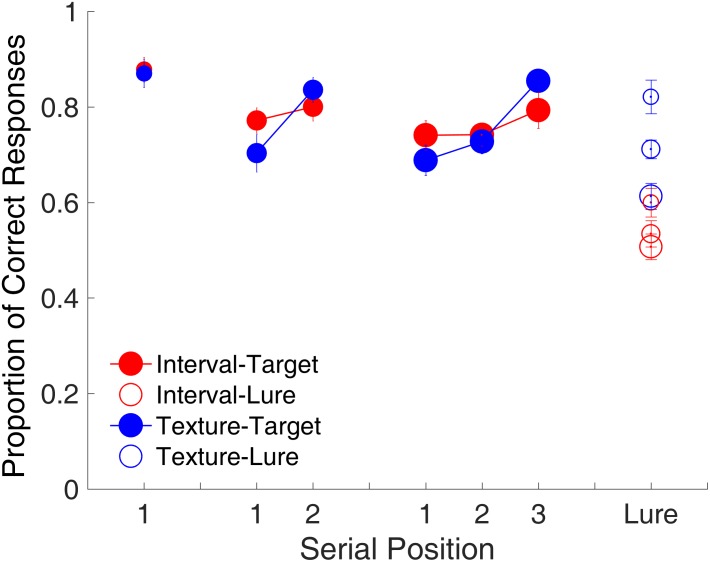
Proportion of correct responses as a function of serial position in Experiment 1. Red and blue represent interval and texture trials, respectively. Filled and open circles represent target and lure trials, respectively. Error bars represent ± 1 standard error of the mean.

**Figure 3** shows the proportion of correct responses plotted separately for each memory load. The serial position effect was examined only for the target trials. For the lure trials, the proportion of correct responses was plotted by collapsing them across memory loads. The memory load of one study item comprised only one serial position. No significant difference was observed in the proportion of correct responses between time intervals and visual textures [*t*_(15)_ = 0.25, *p* = 0.808, Cohen’s *d* = 0.062]. The memory load of two study items comprised Serial Positions 1 and 2. A repeated-measures ANOVA with two factors (serial position and modality) revealed a main effect of serial position [*F*_(1,15)_ = 10.68, *p* = 0.005, $\eta_{p}^{2}$ = 0.416]. The serial position effect differed between time intervals and visual textures, reflected by a significant interaction between serial position and modality [*F*_(1,15)_ = 8.81, *p* = 0.010, $\eta_{p}^{2}$ = 0.370]. Further analyses showed a recency effect only for visual textures, that is, the proportion of correct responses at Serial Position 2 was higher than that at Serial Position 1 [*t*_(15)_ = 3.88, *p* = 0.002, Cohen’s *d* = 0.971]. The memory load of three study items comprised Serial Positions 1, 2, and 3. A repeated-measures ANOVA with two factors (serial position and modality) was conducted, and results indicated a main effect of serial position [*F*_(2,30)_ = 22.20, *p* < 0.001, $\eta_{p}^{2}$ = 0.597] and a significant interaction between serial position and modality [*F*_(2,30)_ = 4.69, *p* = 0.017, $\eta_{p}^{2}$ = 0.238], again showing that the serial position effect differed between the two modalities. Similarly, there was a recency effect between Serial Positions 2 and 3 for visual textures [*t*_(15)_ = 6.73, *p* < 0.001, Cohen’s *d* = 1.681, Bonferroni-corrected], but the same was not observed for time intervals.

For RTs, only a recency effect was found for visual textures between Serial Positions 2 and 3 in the memory load with three study items [*t*_(15)_ = 2.99, *p* = 0.028, Cohen’s *d* = 0.747, Bonferroni corrected]. In addition, RT was around 0.3 s slower for time intervals than for visual textures [*F*_(1,15)_ = 14.71, *p* = 0.002, $\eta_{p}^{2}$ = 0.495, GG corrected] across all positions, as revealed by a repeated-measures ANOVA with two factors (serial position and modality).

#### Similarity Effect

Because similarity increases monotonically with decreasing distance (Kahana and Sekuler, 2002), summed similarity can be determined by the summed probe-study items’ distance (PSD), that is, the summation of the 2D Euclidean distances between the probe and all of the study items (Yotsumoto et al., 2008). The summed similarity is inversely proportional to the summed PSD. To evaluate similarity in trials across different memory loads, the summed PSD was divided by the number of study items to derive the mean PSD. Only lure trials were analyzed for the similarity effect, because it is in lure trials that the probe could be similar to the study items, whereas, in target trials, the probe was identical to one of the study items. All lure trials were sorted based on their mean PSDs. Half of the trials with larger mean PSDs were categorized as small similarity trials, and the other half of the trials with smaller mean PSD were categorized as large similarity trials. It was expected that smaller similarity would lead to more correct rejections of lures as compared with that caused by larger similarity.

**FIGURE 4:**
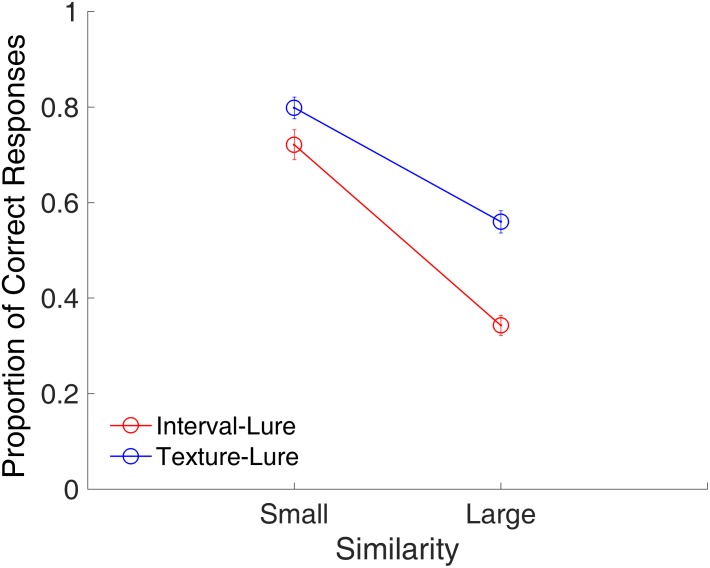
Proportion of correct responses as a function of similarity in Experiment 1. Red and blue represent interval and texture trials, respectively. Error bars represent ± 1 standard error of the mean.

**Figure 4** shows the proportion of correct responses plotted across similarities. A repeated-measures ANOVA with two factors (similarity and modality) indicated main effects of similarity [*F*_(1,15)_ = 252.53, *p* < 0.001, $\eta_{p}^{2}$ = 0.944] and modality [*F*_(1,15)_ = 63.25, *p* < 0.001, $\eta_{p}^{2}$ = 0.808], and a significant interaction between similarity and modality [*F*_(1,15)_ = 20.80, *p* < 0.001, $\eta_{p}^{2}$ = 0.581]. The main effect of similarity showed that the proportion of correct responses was significantly higher for the small similarity condition than for the large similarity condition. The interaction between similarity and modality suggested that the effect of similarity was different between the time intervals and visual textures. Further analysis showed that effect of similarity was larger for time intervals than for visual textures [*t*_(15)_ = 4.56, *p* < 0.001, Cohen’s *d* = 1.141].

#### Binding Effect

In the texture task, the time interval served as the context feature. If there were no binding between the two modalities, the repetition of the interval would not affect the recognition of the visual texture. Similarly, in the interval task, the visual texture served as the context feature. If there were no binding between the two modalities, the repetition of the visual texture would not affect the recognition of the time interval either. We conducted a *post hoc* analysis to evaluate this binding effect.

**FIGURE 5:**
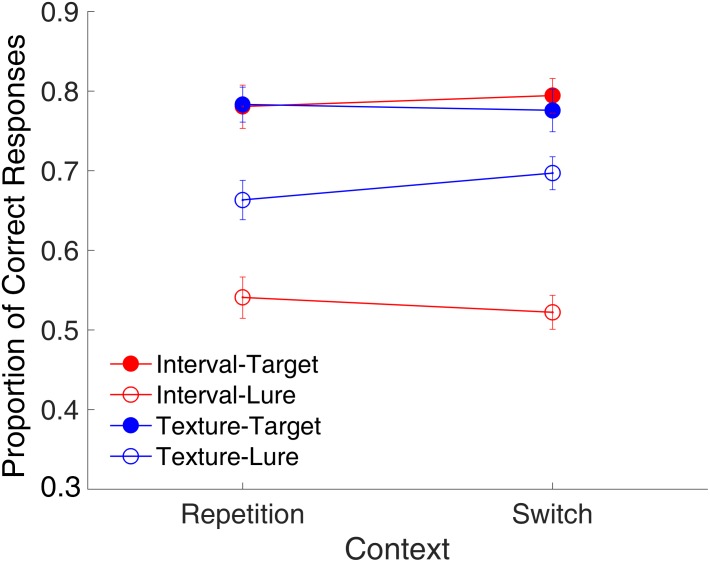
Effects of the context on the proportion of correct responses in Experiment 1. Red and blue represent interval and texture trials, respectively. Filled and open circles represent target and lure trials, respectively. Error bars represent ± 1 standard error of the mean.

**Figure 5** shows the proportion of correct responses plotted across the repetition and switch of the context features. We conducted a repeated-measures ANOVA with three factors (context, modality, and trial type). Results indicated main effects of modality [*F*_(1,15)_ = 48.23, *p* < 0.001, $\eta_{p}^{2}$ = 0.763, GG corrected] and trial type [*F*_(1,15)_ = 38.32, *p* < 0.001, $\eta_{p}^{2}$ = 0.719, GG corrected] a significant interaction of modality and trial type [*F*_(1,15)_ = 17.01, *p* < 0.001, $\eta_{p}^{2}$ = 0.531, GG corrected], and a marginally significant three-way interaction [*F*_(1,15)_ = 4.26, *p* = 0.057, $\eta_{p}^{2}$ = 0.221, GG corrected]. This finding of no main effect of context but a marginal three-way interaction of context, modality, and trial type suggested that the effects of context might differ among the combinations of modality and trial type. Further analysis with a *t*-test only revealed a marginal effect of the repetition of the temporal features on lure textures [*t*_(15)_ = -1.95, *p* = 0.070, Cohen’s *d* = 0.489]. This implies that the repetition or switching of context features might affect the rejection of the lures in different ways for time intervals and visual textures. Further, the same analysis on RTs showed no effect of context (all *p*s > 0.147) for either targets or lures in the two modalities.

### Discussion

Using Sternberg’s item recognition task, we revealed the similar working memory characteristics for time intervals and for visual textures, specifically the effects of memory load and similarity, supporting the hypothesis that time intervals can be represented in working memory as discrete items, similarly, to visual objects. However, the serial position effects differed between interval and texture memory. The serial position effect was only found for visual textures but not for time intervals. In addition, we did not find strong effects of binding.

The memory load effect for time intervals showed a very similar pattern to that for visual textures. This result indicated that working memory for time intervals of visual events is also affected by memory load, as it is for those of auditory events (Teki and Griffiths, 2014; Manohar and Husain, 2016). This is consistent with the memory load effect for time intervals in other paradigms like absolute identification (Lacouture et al., 2001).

The similarity effect was observed for both time intervals and visual textures. By simply dividing the lure trials into the two categories of large similarity (small mean PSD) and small similarity (large mean PSD) trials, we found a similar pattern of similarity effect for both time intervals and visual textures. In Experiment 2, we further investigated the similarity effect by systematically manipulating similarities.

However, the serial position effect, specifically the recency effect, was found only for visual textures. The lack of the primacy effect could be attributed to the short ISIs between the presentations of items (Glenberg et al., 1980). No recency effect was found for time intervals. From the pattern of the data, however, a weak serial position effect for time intervals was observed. Because Experiment 1 contained three memory loads, the number of trials in each memory load condition was constrained, which may not have been adequate for revealing the possible weak recency effect for time intervals. In the next experiments, we investigated the serial position effect in detail.

In addition, we did not find strong evidence for the binding of temporal features and visual features. No effect of the repetition of the visual textures was found on interval memory. On the other hand, there seemed to be a weak effect of the repetition of the temporal features on the rejections of lure textures. Because the studies of feature binding between temporal features and non-temporal features are limited (Bogon et al., 2017), the underlying mechanism remains unclear. One explanation for the weak or absent binding effect may be that task-irrelevant non-spatial features (as temporal and visual features in this study) are weakly bound or not bound to the same events (Hommel, 1998; Chen, 2009). Another explanation might be that feature binding is affected by learning, through which the over-learned feature combinations in long-term memory may facilitate the binding of the same combinations of features (Colzato et al., 2006; Hommel and Colzato, 2009). In Experiment 2, we simplified the visual textures and further examined the effects of binding.

We also found that participants were more biased to judge lure intervals as targets than to judge lure textures, which might lead to the lower proportion of correct responses for lure intervals. In addition, the average RT for time intervals was about 0.3 s larger than that for visual textures. This may be because time is always estimated after visual events (Bogon et al., 2017). In this context, evidence suggests that time intervals may be a high-level stimulus feature encoded at a later stage in the visual processing hierarchy (Maarseveen et al., 2017).

## Experiment 2

In Experiment 2, we further investigated the effects of serial position, as well as similarity and binding, on working memory for time intervals and visual textures, using only the three-item memory load condition. The SDT was incorporated into the recognition task to further investigate the characteristics of the interval memory.

### Materials and Methods

#### Participants

Twelve naïve volunteers from The University of Tokyo (six females, mean age: 25 years, range: 19–32 years) participated in Experiment 2, after excluding three participants because the average accuracy of their recognition of either visual textures or time intervals was at around the chance level, and for one participant, the mean RT for visual textures was 2 standard deviations away from the mean RT across all participants. All participants provided written informed consent for their participation in the experimental protocol, which was approved by the Institutional Review Board of The University of Tokyo. All reported having normal or corrected-to-normal vision.

#### Stimuli and Apparatus

Similar to Experiment 1, 2D compound sinusoidal gratings were used as visual stimuli. Only nine gratings were chosen from the 25 gratings that were used in Experiment 1, with relatively large Euclidian distances from each other, such as 1.51, 1.51; 1.51, 1.75; 2, 1.51; 1.75, 2; 2, 2; 2.25, 2; 2, 2.49; 2.49, 2.25; and 2.49, 2.49 CPD, combined with 9 time intervals derived from a logarithmically spaced range of 0.2512–0.9772 s. The logarithmically spaced setting for time intervals was used to increase the absolute distance between long time intervals so that the distinguishability between long and short time intervals were balanced.

In Experiment 2, three gratings were randomly assigned to three out of nine different spatial frequencies, and three out of nine different time intervals, as study items. The items were labeled as units 1–9 according to their Euclidean distances from the origin of coordinates in spatial frequency [(0, 0) CPD] or in time interval (0 s). One unit PSD meant the distance between two neighbor units, and two units PSD meant the distance between a unit and the unit next to its neighbor, and so on. A probe grating had the following constraints: the PSD between the probe and the target study item was zero, and the PSD between the probe and the non-target study items was at least two units, for either spatial frequencies or time intervals. In addition, the maximum PSD was set to five units, to avoid the location of time intervals at two ends (i.e., the shortest or longest ones). The probe was also set such that it was not equally closest to more than one study item. Finally, we constrained summed PSD to 6, 7, or 8 units for target trials, and 9, 10, or 11 units for lure trials. The same sets of stimuli were used in the interval and texture sessions. The apparatus used in Experiment 2 was the same as that used in Experiment 1.

#### Procedure

**FIGURE 6:**
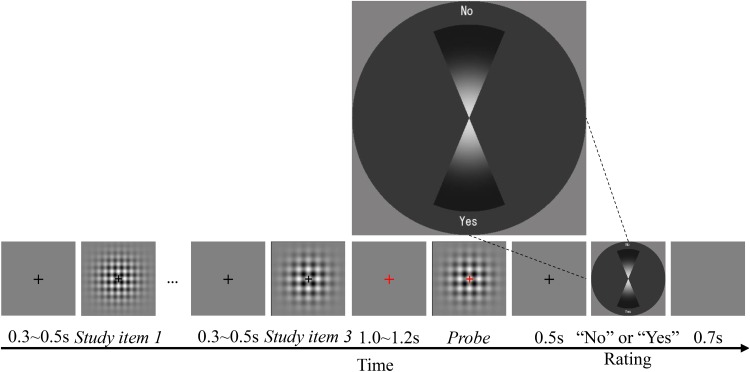
Time course of the recognition/working memory task in Experiment 2.

The procedure for this experiment was the same as for Experiment 1, except that there were always three study items, and the responses were registered using a rating panel (**Figure 6**). A rating panel with a dark gray panel and two vertical sectors representing “Yes” and “No” judgments was presented 0.5 s after the probe disappeared. The participants judged whether the probe was the same as one of the study items and rated their confidence in the judgment on the rating panel. Half of the participants responded “Yes” by clicking on the lower sector, and responded “No” by clicking on the upper sector. The location of the mouse click corresponded to their confidence: the participants were instructed to click further away from the center if they were more confident, and to click closer to the center if they were not very confident. The position of the “Yes” and “No” panels were reversed for the other half of the participants. Participants were asked to respond as quickly as possible, without sacrificing accuracy.

Each session consisted of 18 practice trials and 324 test trials composed of a combination of factors such as serial position (1, 2, or 3), similarity (large, medium, or small), context feature (repetition or switch), and trial type (target trials or lure trials).

### Results

The average accuracy across all available participants for the time intervals was 57.47 ± 3.96% and for the visual textures was 65.26 ± 4.76%; the average RT across all available participants for the time intervals was 1.89 ± 0.51 s and for the visual textures was 1.68 ± 0.28 s.

#### Receiver Operating Characteristic Curve

**FIGURE 7:**
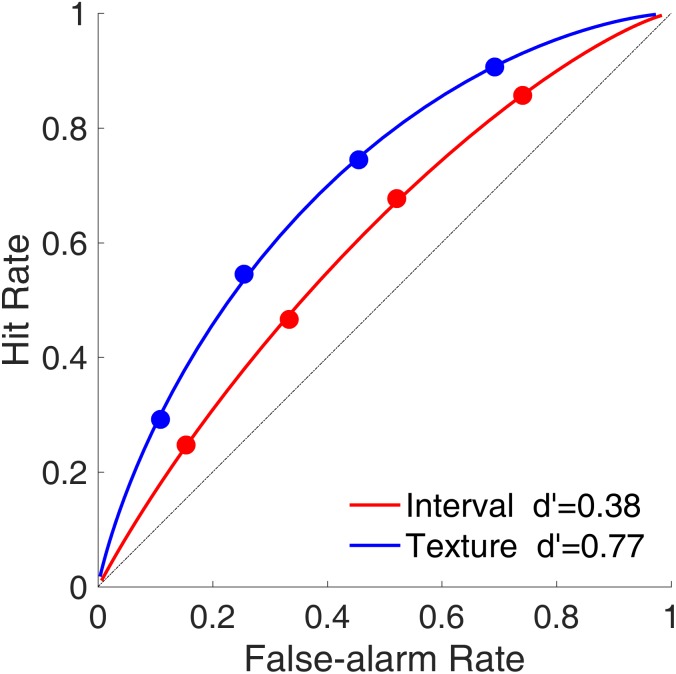
Receiver operating characteristic curves (hit rate as a function of false-alarm rate) in Experiment 2. Red and blue represent interval and texture trials, respectively.

**Figure 7** shows the average ROC curves for the time intervals and visual textures. Despite our intention to equalize task difficulties between the interval session and the texture session, the ROC curves indicated that sensitivity (d′) was smaller for time intervals than for visual textures, indicating that the discriminability between targets and lures was better in the texture task than in the interval task. The curvatures were similar between time intervals and visual textures, which suggested that the shapes of the distributions of the decision criterion (C) for the targets and lures were similar for the two modalities.

#### Serial Position Effect

**FIGURE 8:**
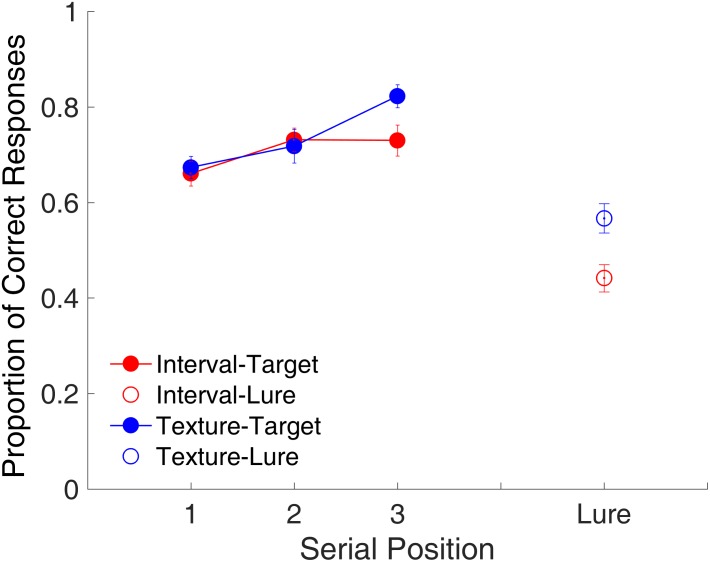
Proportion of correct responses as a function of serial position in Experiment 2. Red and blue represent interval and texture trials, respectively. Filled and open circles represent target and lure trials, respectively. Error bars represent ± 1 standard error of the mean.

**Figure 8** shows the proportion of correct responses plotted across the three serial positions. A repeated-measures ANOVA with two factors (serial position and modality) indicated a main effect of serial position [*F*_(2,22)_ = 14.53, *p* < 0.001, $\eta_{p}^{2}$ = 0.569]. The serial position effect differed between the time intervals and visual textures, with a marginal significance [*F*_(2,22)_ = 3.38, *p* = 0.053, $\eta_{p}^{2}$ = 0.235]. Further analysis indicated the presence of a last-item recency effect for visual textures as observed in Experiment 1, that is, there was a significant difference between Positions 2 and 3 [*t*_(11)_ = -3.06, *p* = 0.032, Cohen’s *d* = 0.884, Bonferroni-corrected]. For time intervals, no significant difference was found between Positions 2 and 3, but there was a significant difference between Positions 1 and 2 [*t*_(11)_ = -2.85, *p* = 0.047, Cohen’s *d* = 0.823, Bonferroni-corrected]. Further, we observed a significant difference between time intervals and visual textures for the lure trials [*t*_(11)_ = -5.05, *p* < 0.001, Cohen’s *d* = 1.458], suggesting that participants were more biased toward judging lure intervals as targets. The ANOVA on RT revealed no significant effects on RT (all *p*s > 0.1).

#### Similarity Effect

**FIGURE 9:**
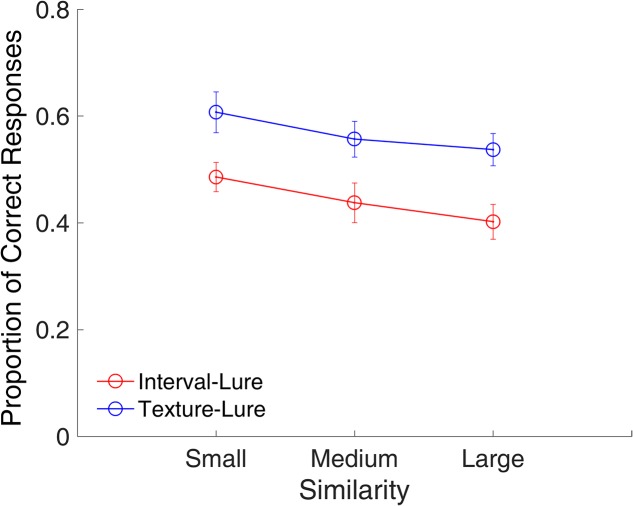
Proportion of correct responses as a function of similarity in Experiment 2. Red and blue represent interval and texture trials, respectively. Error bars represent ± 1 standard error of the mean.

**Figure 9** shows the proportion of correct responses plotted across small, medium, and large similarities. A repeated-measures ANOVA with two factors (similarity and modality) indicated a main effect of similarity [*F*_(2,22)_ = 13.31, *p* < 0.001, $\eta_{p}^{2}$ = 0.548] and modality [*F*_(1,11)_ = 25.32, *p* < 0.001, $\eta_{p}^{2}$ = 0.697], indicating that the participants were more likely to consider a lure interval as a target as compared to a lure texture.

#### Binding Effect

**FIGURE 10:**
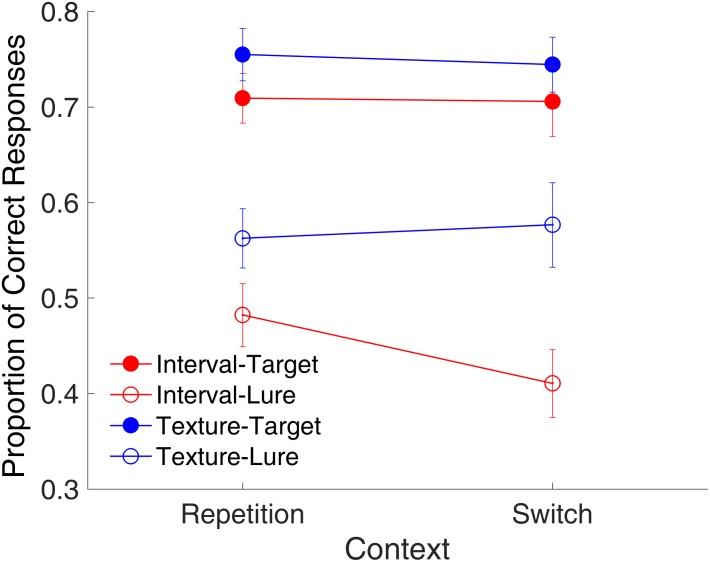
Proportion of correct responses as a function of context in Experiment 2. Red and blue represent interval and texture trials, respectively. Filled and open circles represent target and lure trials, respectively. Error bars represent ± 1 standard error of the mean.

**Figure 10** shows the proportion of correct responses plotted across the repetition and switch of the context features. Again, we conducted a *post hoc* analysis on the binding effect. Unlike Experiment 1, in Experiment 2, it was possible that, when both the temporal feature and visual feature of the probe matched the features of the study items, the two features did not belong to the same study item. Therefore, we selected trials in which the two features belonged to the same study item. We conducted a repeated-measures ANOVA with three factors (context, modality, and trial type). Results indicated main effects of modality [*F*_(1,11)_ = 15.64, *p* = 0.002, $\eta_{p}^{2}$ = 0.587] and trial type [*F*_(1,11)_ = 29.37, *p* < 0.001, $\eta_{p}^{2}$ = 0.728]. Although there were no main effects of context and no significant interactions between context and other factors, further analysis with a *t*-test indicated a significant difference between the repetition and switching of context textures for the lure intervals [**Figure 10**, *t*_(11)_ = 2.24, *p* = 0.0467, Cohen’s *d* = 0.647]. The same analysis on RTs showed no effects of context (all *p*s > 0.592).

### Discussion

By combining the recognition task with a signal detection task, we provided further evidence to support the idea that time intervals can be stored in and retrieved from working memory as discrete items. The ROC curves showed similar curvatures between time intervals and visual textures, suggesting similar distributions of C for judging temporal and visual items. The serial position effect was found for time intervals, a robust similarity effect was observed again, but a strong binding effect was still not found. We also confirmed that lures in the interval memory were more likely to be misperceived as targets.

Although we did not observe a clear last-item recency effect for time intervals, we still found the serial position effect for the interval memory. According to a two-component account (Phillips and Christie, 1977; Allen et al., 2014), the absence of a last-item recency effect may be attributed to the interference of the last item by subsequent events. In the present experiment, the rating panel may have disrupted the recency effect for time intervals because participants had to make a relatively complicated decision regarding rating their confidence during the judgment. The absence of a recency effect was also seen in some conditions in a study by Manohar and Husain (2016). They claimed that recency effects are susceptible to attentional disruption by the presence of irrelevant information.

After carefully manipulating the similarity between probe and study items, we observed a robust similarity effect, as also observed in Experiment 1, for both time intervals and visual textures. These results confirm that time intervals can be stored as discrete but noisy items, similarly, to visual working memory, and that working memory for temporal items is also influenced by temporal similarities between the items.

## Experiment 3

In Experiment 3, we further examined the memory characteristics of the longer durations of the supra-second range. We used the same recognition task as that used in Experiment 1, with only three study items, to examine the characteristics of working memory only for time intervals in the sub- with the supra-second range. We mixed and randomized the trials in the two ranges in each session. In this experiment, we only measured interval memory.

### Materials and Methods

#### Participants

Ten naïve volunteers from The University of Tokyo (three males, mean age: 20 years, range: 19–21 years) participated in Experiment 3, after excluding two participants because their overall accuracy for both gratings and time intervals was around the chance level.

#### Stimuli and Apparatus

2D gratings were presented with different time intervals in either a sub- or supra-second range. The spatial frequencies of the 2D gratings were at the same Euclidian distance from the origin as (2, 2) CPD, but with five different radians from a linear spaced range of pi/2^∗^5/16 to pi/2^∗^11/16. Subsequently, the gratings were of 2D spatial frequencies such as 1.33, 2.49; 1.68, 2.27; 2, 2; 2.27, 1.68; and 2.49, 1.33 CPD. In this way, only the phases (the apparent texture) but not the distances were different, reducing the variability between visual textures.

For the time intervals, we first randomly selected 15 intervals from the sub-second range of 0.5–0.7 s and another 15 from the supra-second range of 2–2.8 s. We then derived a set of five intervals by adding 0.2 ^∗^ (-2, -1, 0, 1, 2) s to each of the selected intervals. The target study item was the middle interval after adding the 0. The other two non-target study items were selected from the remaining four intervals. In the target trials, the probe matched the target study item, and in the lure trials, the probe was selected from the two remaining unselected intervals.

The apparatus used in Experiment 3 was the same as that used in Experiments 1 and 2.

#### Procedure

As shown in **Figure 1**, the procedure of Experiment 3 was the same as that of Experiment 1, except for the use of the memory load of three study items only. Each participant completed one session with mixed trials in either the sub- or supra-second range. The time intervals were in the same range within each trial. There were 18 practice trials and 360 test trials composed of a combination of factors such as time range (sub- or supra-second), serial position (1, 2, or 3), context (repetition or switch), and trial type (target trials or lure trials).

### Results

The average accuracy across all available participants for the sub-second intervals was 61.68 ± 4.45% and for the supra-second intervals was 63.54 ± 4.36%; the average RT across all available participants for the sub-second intervals was 0.79 ± 0.32 s and for the supra-second intervals was 0.86 ± 0.36 s.

#### Serial Position Effect

**FIGURE 11:**
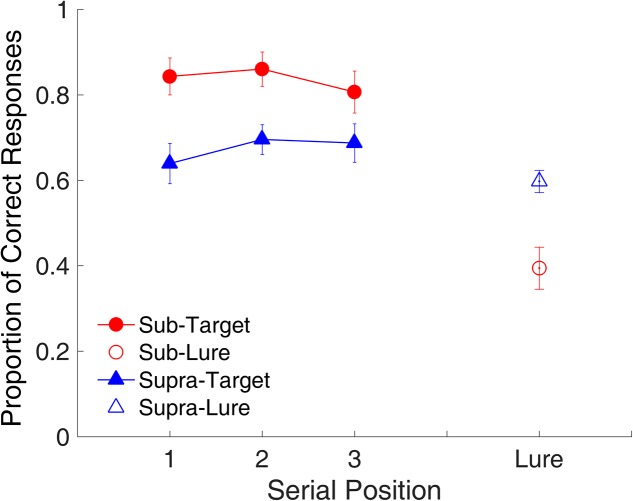
Proportion of correct responses as a function of serial position in Experiment 3. Red and blue represent sub- and supra-second trials, respectively. Filled circles and triangles represent target trials, and open circles and triangles represent lure trials. Error bars represent ± 1 standard error of the mean.

**Figure 11** shows the proportion of correct responses plotted across serial positions. A repeated-measures ANOVA with two factors (serial position and modality) indicated no main effect of serial position [*F*_(2,18)_ = 1.22, *p* = 0.319, $\eta_{p}^{2}$ = 0.119], but a main effect of time range [*F*_(1,9)_ = 12.63, *p* = 0.006, $\eta_{p}^{2}$ = 0.584], indicating that the proportion of correct responses was significantly higher for the sub- than for the supra-second interval. The ANOVA on RTs indicated that the RT was significantly lower [*F*_(1,9)_ = 9.59, *p* = 0.013, $\eta_{p}^{2}$ = 0.516] for the sub- than for the supra-second interval.

#### Similarity Effect

To compare the stages of processing of between the sub- and supra-second intervals, we examined how similarity affected the d′ and C pertaining to the “Yes/No” judgment.

**FIGURE 12:**
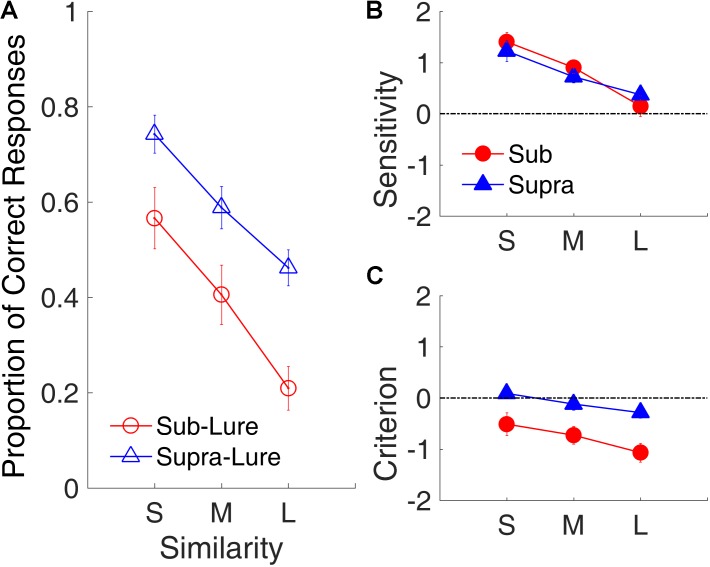
Similarity effect in Experiment 3. **(A)** Proportion of correct responses as a function of similarity. Open circles and triangles show lure trials. **(B)** d′ as a function of similarity. **(C)** C as a function of similarity. Red and blue represent sub- and supra-second trials, respectively. Error bars represent ± 1 standard error of the mean.

**Figure 12** shows the effects of similarity on the proportion of correct responses, d′ and C. We conducted a repeated-measures ANOVA with two factors (similarity and modality) for each performance measure. The results indicated robust similarity effects on the proportion of correct responses [*F*_(2,18)_ = 24.79, *p* < 0.001, $\eta_{p}^{2}$ = 0.734], d′ [*F*_(2,18)_ = 16.07, *p* < 0.001, $\eta_{p}^{2}$ = 0.641, GG corrected], and C [*F*_(2,18)_ = 10.88, *p* < 0.001, $\eta_{p}^{2}$ = 0.547]. Further, we observed significant effects of time range on the proportion of correct responses [*F*_(1,9)_ = 20.21, *p* = 0.001, $\eta_{p}^{2}$ = 0.692] and C [*F*_(1,9)_ = 19.68, *p* = 0.002, $\eta_{p}^{2}$ = 0.686], indicating that participants were more likely to judge lures in the sub-second intervals as targets than to judge lures in the supra-second intervals as targets. The difference in decision criterion (C) instead of in the sensitivity (d′) between the two time ranges suggests that the difference between the processing of the sub- and of supra-second intervals might occur in the decision-making stage.

#### Binding Effect

**FIGURE 13:**
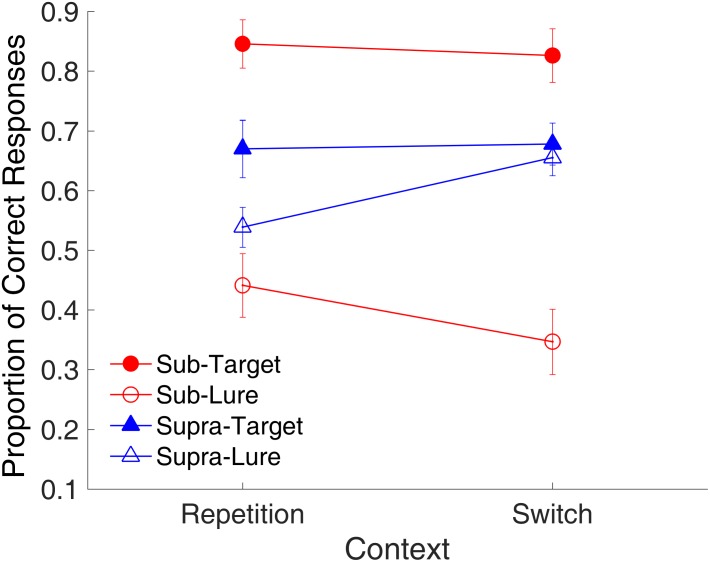
Effects of context on proportion of correct responses in Experiment 3. Red and blue represent sub- and supra-second trials, respectively. Filled circles and triangles show target trials, and open circles and triangles show lure trials. Error bars represent ± 1 standard error of the mean.

**Figure 13** shows the effect of context on the proportion of correct responses. A repeated-measures ANOVA with three factors (context, time range, and trial type) was conducted. Results indicated a main effect of trial type [*F*_(1,9)_ = 17.89, *p* = 0.002, $\eta_{p}^{2}$ = 0.665, GG corrected]; two-way significant interactions of context and time range [*F*_(1,9)_ = 6.80, *p* = 0.028, $\eta_{p}^{2}$ = 0.431, GG corrected], and of time range and trial type [*F*_(1,9)_ = 20.95, *p* = 0.001, $\eta_{p}^{2}$ = 0.700, GG corrected]; and a significant three-way interaction of context, time range, and trial type [*F*_(1,9)_ = 5.57, *p* = 0.043, $\eta_{p}^{2}$ = 0.382, GG corrected].

Similar to the binding effect observed in Experiment 1, the significant three-way interaction suggested that the effects of context differed among the combinations of modality and trial type. Further analysis with a *t*-test showed a marginal difference between the repetition and switch of the context [*t*_(9)_ = 2.22, *p* = 0.054, Cohen’s *d* = 0.703] for the lure sub-second intervals, consistent with the findings of Experiment 2. This result suggested that the repetition of visual textures facilitated the correct rejection of the lure intervals, however, for supra-second intervals, the repetition increased the false-alarm rate [*t*_(9)_ = -3.30, *p* = 0.009, Cohen’s *d* = 0.845].

The same analysis on RT showed the main effect of trial type [*F*_(1,9)_ = 22.44, *p* = 0.001, $\eta_{p}^{2}$ = 0.714, GG corrected]; two-way significant interactions of context and time range [*F*_(1,9)_ = 13.80, *p* = 0.005, $\eta_{p}^{2}$ = 0.605, GG corrected], of context and trial type [*F*_(1,9)_ = 9.41, *p* = 0.013, $\eta_{p}^{2}$ = 0.511, GG corrected], and of time range and trial type [*F*_(1,9)_ = 6.76, *p* = 0.029, $\eta_{p}^{2}$ = 0.429, GG corrected]. The repetition of the visual textures facilitated recognition of the target sub-second intervals by reducing the RT [*t*_(9)_ = -2.74, *p* = 0.023, Cohen’s *d* = 0.866]; for supra-second intervals, however, the repetition increased the RT [*t*_(9)_ = 9.03, *p* < 0.001, Cohen’s *d* = 0.720].

### Discussion

Using the temporal recognition/working memory task, we compared the effects of serial position, similarity, and binding for time intervals in the sub-second range with those in the supra-second range. The effect of similarity was observed for both ranges. Specifically, the repetition of context features had opposite effects between the two ranges, but no serial position effect was found for either range of time intervals.

The absence of the serial position effect for supra-second intervals was consistent with the findings of Teki and Griffiths (2014). On the other hand, for sub-second intervals, the present finding was not consistent with the results of Experiment 2, where serial position effect was observed. One reason might be the mixing of the supra- and sub-second intervals in a single session. Specifically, the brain network recruited for the processing of supra-second intervals in previous trials may affect the processing of subsequent peri-second intervals (Murai and Yotsumoto, 2016a). Therefore, the mixing might have led to an interference with the processing of peri-/sub-second intervals such that it disrupted the serial position effect for sub-second intervals. It is difficult to draw strong conclusions from the present evidence, and the absence of recency effects must be interpreted with caution. Further research is necessary to clarify this issue.

The difference of processing of between sub- and supra-second intervals may occur in the later stage of processing. Previous studies on the similarity effect showed that participants are less likely to judge a probe as one of the study items when the similarity among the study items are high (Kahana and Sekuler, 2002; Nosofsky and Kantner, 2006; Viswanathan et al., 2010). In our experiment, the supra-second intervals may be less distinctive, as they are perceived less precisely (Murai and Yotsumoto, 2016b; but see Lewis and Miall, 2009) as compared to sub-second intervals. This predicts that sub-second intervals are more likely to be judged as the targets, as was the case in the present study. The difference in this bias might occur in the decision-making stage instead of during sensory processing, as reflected by the difference in the decision criterion (C) instead of the sensitivity (d′) between the sub- and supra-second intervals.

Further, the difference in the proportion of correct responses between the repetition and switching of the context features was found only for the lure intervals in both ranges, but in the opposite way. Specifically, the repetition of visual textures led to a higher correct rejection of lure sub-second intervals but a lower correct rejection for lure supra-second intervals, as compared with that caused by the switching of visual textures. If there is indeed a binding and the retrieval of previous bindings occurs, according to Bogon et al. (2017), the partial repetition should lead to worse recognition, and it was so for the lure trials in the supra-second intervals. However, the same was not observed for sub-second intervals. No context effect was found for target intervals, that is, the repetition/switch of visual textures did not seem to contribute to the recognition of target intervals.

## General Discussion

Using Sternberg’s item recognition task and framework of SDT, we examined working memory for multiple time intervals, and compared its characteristics, including the effects of memory load, serial position, and similarity, with that for visual textures. In Experiment 1, we observed the same patterns of memory load effect and similarity effect between time intervals (in sub-second range) and visual textures, supporting the hypothesis that, similarly, to visual objects, time intervals are represented in working memory as discrete items. However, serial position effect was absent for the interval memory. In Experiment 2, we further investigated serial position effect using a rating scale. We found a serial position effect for the time intervals, although it was weak and slightly different from that for visual textures, further supporting our hypothesis. In Experiment 3, we examined interval memory of the supra-second range, and found the supra-second intervals might also be represented as discrete items, although there were differences between working memory for the sub- and that for the supra-second intervals.

### Item Representations of Time Intervals in Working Memory

Working memory for multiple time intervals can be compared to working memory for multiple visual objects. Visual working memory has a limited storage, and memory performance is influenced by the number of study items, which yields a memory load effect (Luck and Vogel, 1997; Bays and Husain, 2008). The memory load effect for time intervals showed a similar pattern as that for visual textures, especially for targets that had already been stored in working memory. This finding suggests that, similar to visual textures, time intervals can be represented as discrete items in working memory, and the countable number of temporal items influences temporal working memory. The memory load effect for time intervals has also been observed in previous studies with auditory stimuli (Lacouture et al., 2001; Teki and Griffiths, 2014; Manohar and Husain, 2016). However, these studies did not directly compare temporal working memory with auditory working memory. Therefore, future studies should make direct comparisons and confirm whether the working memory representation of time intervals can be similar to that of auditory or other non-temporal items, as to that of visual items.

According to the noisy exemplar model (Kahana and Sekuler, 2002), visual stimuli are represented as separate but noisy items in working memory, and the recognition of visual items is influenced by the similarity between the probe and study items; time intervals may be represented in the same way. Our results showed the same patterns of the similarity effect for lure intervals as for lure textures, and thus confirmed that the item representations of time intervals were similar to those of visual textures. The similarity effect for visual items has been well investigated in previous studies (e.g., Kahana and Sekuler, 2002; Zhou et al., 2004; Yotsumoto et al., 2008), but that for temporal items was examined for the first time in the present study.

The noisy-item representations of time intervals are further supported by the findings pertaining to the ROC curve. A regular and symmetric ROC curve implied the probability distributions of the decision criterion (C) for targets and lures. The similar curvature of the ROC curves found in our study indicates that the C distributions for time intervals share common properties with those for visual textures. Although the C distributions for target intervals and lure intervals may overlap, target intervals can be distinguished from lure intervals according to a specific C. The noisy exemplar model proposed that the recognition memory of items is also affected by the inter-study item similarity (Kahana and Sekuler, 2002). Therefore, future studies need to examine this proposal with reference to temporal items.

The item representations of time intervals are of some difference from those of visual objects. Although we found in Experiment 2 that the later presented intervals were recognized better than were those presented earlier, consistent with the definition of the recency effect, this recency effect was different from the last-item recency effect observed for visual textures. Further, we observed the absence of the serial position effect for sub-second intervals in Experiments 1 and 3, and for supra-second intervals in Experiment 3. Traditionally, there are two possible accounts for the recency effects for visual items. The first is the two-component (Phillips and Christie, 1977; Allen et al., 2014) based dual-store model, which proposes that the later presented items are stored in an active but limited-capacity buffer. In this account, attention is allocated to the current presented item, and it is switched from the earlier item to the later item. Therefore, the later presented items are retrieved better than the earlier ones are, as they are no longer in the buffer but are instead likely to be in long-term storage. This account cannot explain the weak or even absent recency effect for temporal items. The other account, the distinctiveness-based single-store model (Neath, 1993), stresses the role of temporal distinctiveness between the study items. According to this model, later presented items are relatively more distinctive at retrieval and are therefore retrieved more easily. This account cannot explain the absence of the last-item recency effect for temporal items either.

A possible explanation may be that the item representations of time intervals are weaker than those for visual objects are. In this case, recognition relies more on perceptual averaging across sequential items, which would ignore the serial positions of items, than on the identities of individual items (Ariely, 2001; Zhou et al., 2015). Another possible explanation may be related to the dual role of time intervals. Time intervals of events are not only the contents *per se*, but they are also the temporal framework for the contents of events (e.g., visual textures in the study) stored in and retrieved from recognition memory (Fournier and Gallimore, 2013; Thavabalasingam et al., 2016; Bogon et al., 2017).

Another difference is that lure items in the sub-second range are more likely to be misjudged as target items as compared with lure items in the visual modality. At the same time, the judgment for target items in both modalities yielded very similar performances. It is not clear why the hit rates were similar while the false-alarm rates were discrepant between time intervals and visual textures. In the SDT framework, both the difference of d′ and C would lead to these results. Future studies need to control d′ between the interval and texture tasks to examine whether this tendency occurred due to C, and thus in the decision-making stage, or whether this tendency is related to the domain-general working memory processes, regardless of the modality.

### Binding and the Mechanism Underlying the Item Representations of Time Intervals

The mechanisms underlying the item representations for the temporal and visual modalities may share common properties. As for visual objects, the neural object-file theory proposes that objects are first individualized to discrete items, named as “object files” (Kahneman et al., 1992). However, at the first stage of processing, item representations are not sufficient to be recognized, and it is at the second stage of processing that the binding of features into the object files make the objects distinctive, such that they can be recognized as targets or lures (Xu and Chun, 2009). There may be a similar mechanism for the individualization and recognition of time intervals as discrete items in working memory.

Temporal and visual features belonging to the same events might interact with each other because of the binding. Our results suggested the presence of possible binding effects between visual memory and interval memory. In our study, the effects were only seen on lures but not on targets. It may be because the task-irrelevant feature is more weakly bound to the event as compared with the task-relevant feature. Additionally, the repetition of the temporal feature led to worse recognition performance for the visual features, consistent with that for the auditory feature (Bogon et al., 2017). The mechanism underlying these findings is not yet clear, and therefore, more studies need to examine the binding of temporal and non-temporal features.

### Difference Between Working Memory for Sub- and Supra-Second Intervals

Time intervals in sub- and supra-second ranges might be retrieved differently. In our study, the difference between the recognition of sub- and supra-second intervals was characterized by the difference in the value of C. Sensitivity (d′) was similar for both ranges, meaning that the early processing of the two ranges of time intervals were similar. The difference in C between the sub- and supra-second intervals suggests that the difference in working memory for the two ranges of time intervals may occur in the later stage of processing, like in the decision-making stage. Further, sub-second intervals tended to be judged as the targets more often as compared to supra-second intervals. Previous studies suggested that hierarchical timing networks are recruited for processing time intervals in two ranges, with lower-level processes affecting both ranges but having a stronger effect on sub-second intervals. Further, higher-level processes have been found to govern both ranges but have a stronger effect on supra-second intervals (Wiener et al., 2010; Murai and Yotsumoto, 2016b; Petter et al., 2016).

As for the neural mechanisms underlying interval perceptions of sub- and supra-seconds, a number of studies have reported distinct but partially overlapped mechanisms for those two ranges (Wiener et al., 2010; Murai and Yotsumoto, 2016b; Petter et al., 2016). The cerebellum has been reported to be involved in both sub- and supra-second timing (for humans, Petter et al., 2016; for money, Ohmae et al., 2017). Supra-second intervals (e.g., >2,000 ms) have been reported as being further processed by the cortico-thalamic-striatal circuit (Petter et al., 2016). The shared neural mechanisms (e.g., cerebellum) could be a part of the shared earlier stage of processing, and the neural networks specific to the supra-second intervals (e.g., the cortico-thalamic-striatal circuit) could be the level that differentiates the decision stage (C).

### Limitations and Other Implications

There were several limitations to this study. First, we failed to equate the task difficulties between modalities, resulting in different d′ between the interval and texture tasks. Although we intended to equalize difficulties, this was challenging because of the relatively large individual differences and interactions between conditions. The second limitation was that the highest memory load was limited to three. Thus, we could not provide a comparison of the memory load effect between time intervals and visual objects under a broader range of conditions (Luck and Vogel, 1997; Cowan, 2001). Thereby, we could not provide evidence for clarifying the ongoing debate between the two competing views of working memory capacity (Luck and Vogel, 1997; Bays and Husain, 2008). Third, we did not use a unified set of stimuli for all three experiments. Instead, we changed the stimulus design across three experiments. Regardless of these limitations, this study revealed the following important characteristics of interval memory: the working memory representation of time intervals is similar to that of visual objects, albeit with some differences between the two modalities.

While interval perception has been examined in various populations, such as young healthy adults, children (e.g., Clement and Droit-Volet, 2006; Droit-Volet and Rattat, 2007; Rattat and Droit-Volet, 2007; Droit-Volet, 2008, 2017; Droit-Volet and Izaute, 2009; but see Droit-Volet et al., 2007, for short-term memory of single interval), older adults (e.g., McCormack et al., 1999, 2002; Lustig and Meck, 2001; Bherer et al., 2007; Lustig and Meck, 2011; Anderson et al., 2014), and clinical patients (e.g., mild cognitive impairment: Rueda and Schmitter-Edgecombe, 2009; Heinik, 2012; Mioni et al., 2018; Alzheimer’s disease: Nichelli et al., 1993; Hellstrom and Almkvist, 1997; Carrasco et al., 2000; Caselli et al., 2009; El Haj et al., 2013, 2014), the working memory aspects of multiple intervals have been left uninvestigated. When it applies to real life situations, working memory always takes place in our timing and time perception. We believe that further studies in working memory representations of interval times will provide more insights into the neural and psychological properties of interval perception for various populations.

## Conclusion

By comparing working memory for time intervals with that for visual textures, we showed the similar characteristics of temporal and visual working memory, specifically with reference to the effects of memory load, serial position, and similarity. We also found some differences in temporal and visual working memory. Specifically, the effect of serial position was weaker or even absent for interval memory. Further, time intervals in the sub-second range were more likely to be judged as remembered in working memory than were visual textures. In this study, we demonstrated that multiple time intervals are represented in working memory as discrete items, and that working memory for time intervals in the sub- and supra-second range may differ in the later stage of processing.

## Author Contributions

ZF and YY conceived and designed the experiments and wrote the manuscript. ZF performed the experiments and analyzed the data.

## Conflict of Interest Statement

The authors declare that the research was conducted in the absence of any commercial or financial relationships that could be construed as a potential conflict of interest.
